# Telomerase Immortalization of Human Corneal Endothelial Cells Yields Functional Hexagonal Monolayers

**DOI:** 10.1371/journal.pone.0051427

**Published:** 2012-12-21

**Authors:** Thore Schmedt, Yuming Chen, Tracy T. Nguyen, Shimin Li, Joseph A. Bonanno, Ula V. Jurkunas

**Affiliations:** 1 Schepens Eye Research Institute, Massachusetts Eye and Ear Infirmary, Department of Ophthalmology, Harvard Medical School, Boston, Massachusetts, United States of America; 2 School of Optometry, Indiana University, Bloomington, Indiana, United States of America; University of Florida, United States of America

## Abstract

Human corneal endothelial cells (HCEnCs) form a monolayer of hexagonal cells whose main function is to maintain corneal clarity by regulating corneal hydration. HCEnCs are derived from neural crest and are arrested in the post-mitotic state. Thus cell loss due to aging or corneal endothelial disorders leads to corneal edema and blindness–the leading indication for corneal transplantation. Here we show the existence of morphologically distinct subpopulations of HCEnCs that are interspersed among primary cells and exhibit enhanced self-renewal competence and lack of phenotypic signs of cellular senescence. Colonies of these uniform and hexagonal HCEnCs (HCEnC-21) were selectively isolated and demonstrated high proliferative potential that was dependent on endogenous upregulation of telomerase and cyclin D/CDK4. Further transduction of HCEnC-21 with telomerase yielded a highly proliferative corneal endothelial cell line (HCEnT-21T) that was devoid of oncogenic transformation and retained critical corneal endothelial cell characteristics and functionality. This study will significantly impact the fields of corneal cell biology and regenerative medicine.

## Introduction

The cornea is composed of three main cell types–epithelium, stromal keratocytes, and endothelium–each with unique properties to maintain corneal clarity and to contribute to the main refractive element of the eye. Human corneal endothelium (HCEn) is a monolayer of hexagonal cells situated in the posterior surface of the cornea and has the key function of maintaining appropriate corneal hydration, necessary for clear vision. HCEn retains corneal clarity by providing a barrier function between the corneal stroma and aqueous humor, and by active ion transport mechanisms, which balance the swelling pressure of the cornea. HCEn is arrested in the post-mitotic state and does not proliferate in vivo [1]. Age- and disease-related loss of human corneal endothelial cells (HCEnCs) is a major cause of corneal blindness and the most common cause for corneal transplantation in the US.

HCEn is derived from cranial neural crest cells (subsequently mesenchymal cells) whose migration from the margins of the optic cup is triggered by the separation of lens vesicle from surface ectoderm [2]. Although initially a double layer, HCEn becomes a single layer of flattened hexagonal cells that rests on its basal lamina, Descemets membrane, and starts forming apical-basal polarization and apical tight junctions, which characteristically persist throughout adult life. Several reports on the existence of endothelial progenitor cells situated in the peripheral cornea have not been confirmed. Refutability of the existence of corneal endothelial progenitor cells in the adult population is supported by the very low proliferative potential and limited passaging ability of HCEn in vitro, rapid cellular senescence, and eventual endothelial-to-mesenchymal transition (EMT) [3], [4], [5]. EMT is a pathophysiologic mechanism resulting in fibroblast-like transformation and loss of the endothelial-specific cell phenotype that is frequently observed in pathologic conditions and in primary HCEn cell cultures [6]. To date, we do not know how to generate uniform and functional corneal endothelial monolayers from stem cells or other cell types and corneal tissue remains the only predictable source of HCEnCs. However, use of this tissue has significant drawbacks due to limited mitotic capacity and loss of characteristic morphology in vitro, which, in turn, hamper development of disease models and regenerative cell therapies.

Previous investigations aiming at establishing long-term cultures of HCEnCs relied solely on oncogenic manipulation of HCEnCs, for example, transformation using the viral oncogenes SV40 large T antigen and HPV E6/E7 or overexpression of mutant CDK4 [7], [8], [9], [10]. Viral oncogenes are well known to abrogate the p53 pathway, which strongly interferes with studies on stress-related mechanisms and apoptosis, both of which have been of special interest to endothelial cell biologists studying common corneal endothelial disorders such as Fuchs dystrophy [11]. In addition, mutant CDK4-expressing HCEnCs lost the crucial corneal endothelial cell morphology and tight junction formation, thus bringing into question their usefulness as a model system to study HCEnCs. In contrast, human telomerase reverse transcriptase (hTERT) expression has been shown to be effective in extending the life span of various cell types, with minimal impact on cell physiology and differentiation state; however, the role of hTERT in immortalization of HCEnCs has not been explored in the past [12], [13].

The hTERT catalytic subunit has been employed extensively to extend the life span of a variety of human cell types, because its expression is not accompanied by cancer-associated changes or chromosomal abnormalities [14]. However, whether or not hTERT alone would be sufficient to immortalize a certain cell will depend on tissue-specific characteristics and mitotic competence. Bodnar et al. were the first to describe immortalization of foreskin fibroblasts and retinal pigment epithelial cells by introduction of the hTERT catalytic subunit [15]. Similarly, different types of mitotically competent somatic cells such as epithelial cells [16], [17] and vascular endothelial cells [18] have been found to be responsive to hTERT expression, leading to the development of proliferative and phenotypically specific cell lines. In contrast, immortalization with hTERT alone has been problematic in mitotically incompetent somatic cells such as neural, glial, and muscle cells, thus immortalization with hTERT has only been reported for their replicating progenitors, not for the terminally differentiated cells themselves [19]. HCEn is another example of terminally differentiated somatic cells that, like neuronal tissue, are neural crest-derived and mitotically arrested. Moreover, there is a growing need to generate corneal endothelial cell lines to study disease processes, especially premature depletion of cells in vivo, resulting in corneal blindness. The development of reliable and long-lasting cell culture systems is of eminent importance to provide better models for the study of HCEnC biology and regeneration. In this manuscript, we investigated whether hTERT expression alone is sufficient to immortalize human corneal endothelial cells. We detected that primary endothelial cell cultures exhibit distinct subpopulations of endothelial cells that, after isolation, were conducive to hTERT immortalization. A highly uniform subpopulation of endothelial cells (HCEnC-21) was derived from primary cells harvested from a 21-year-old male (21M) donor. Following transduction with hTERT, HCEnC-21 yielded highly hTERT-expressing cells (HCEnC-21T). To our knowledge, this is the first report of a corneal endothelial cell immortalization that is not based on oncogene expression, and that is able to simultaneously preserve high proliferative activity, as well as corneal endothelial morphology, marker characteristics, and functionality.

## Results

### Corneal Endothelial Primary Cell Cultures Consist of Different Subpopulations of Cells

**Figure 1:**
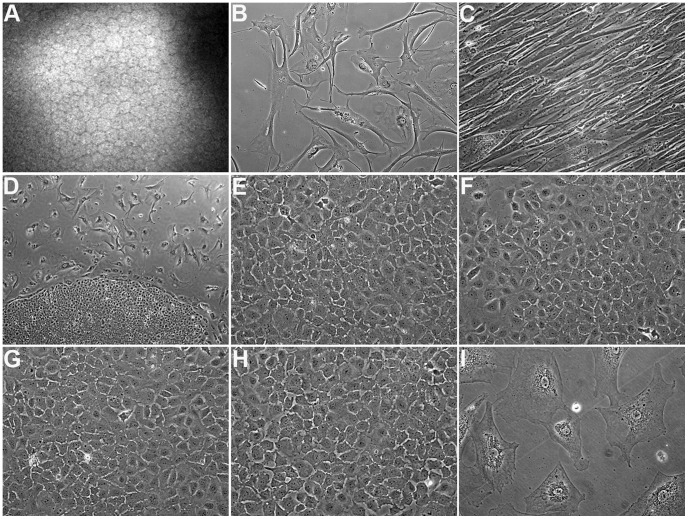
Morphologic study of corneal endothelial primary cells. (A) In vivo confocal microscopy of human corneal endothelium demonstrating the typical hexagonal cell morphology. (B) Phase-contrast micrograph of non-dividing primary cells from a 21-year-old donor (21M) at passage 9, displaying typical signs of senescence. 200x. (C) Phase-contrast micrograph of primary cells from a 63-year-old donor that underwent endothelial-mesenchymal transformation at passage 1. Note that the cells lost the typical corneal endothelial morphology and appear fibroblast-like. 200x. (D) Phase-contrast micrograph showing 2 morphologically distinct subpopulations of cells in the 21M primary culture. The uniform cells growing in colony-like structures were designated HCEnC-21. 40x. (E-H) Phase-contrast micrographs of HCEnC-21 at passages 24 (E) and 46 (F) as well as telomerase-transduced HCEnC-21 (HCEnC-21T) at passages 25 (G) and 50 (H). Importantly, HCEnC-21 and HCEnC-21T cells grew in contact-inhibited monolayers displaying the typical hexagonal cell morphology seen in vivo. 200x. (I) Phase-contrast micrograph of telomerase-transduced 21M (21M+hTERT) at passage 7 showing a senescent phenotype. 200x.

The HCEn is a monolayer of highly uniform cells with characteristic hexagonal morphology that are non-proliferative in vivo (Figure 1A). HCEnCs can be isolated and cultured in vitro; however, their proliferative potential is very low, and primary HCEnCs show a strong tendency to enter senescence. Figure 1B shows a phase-contrast micrograph of passage-9 primary HCEnCs. These were isolated from a 21-year-old donor (21M) and displayed typical signs of senescence, e.g. large and flat morphology, binucleation, and granulation. In addition, even early-passage primary HCEnCs are highly prone to EMT, which changes the cell phenotype into an elongated and fibroblast-like morphology. Figure 1C depicts primary HCEnCs isolated from a 63-year-old donor that underwent EMT at passage 1 resulting in the loss of corneal endothelial-specific hexagonal morphology and transformation into an elongated and fibroblast-like abnormal phenotype. To establish corneal endothelial long-term cell cultures that retain corneal endothelial characteristics, the morphological differences among cells in a series of primary cultures from different donor corneas were investigated. Among largely non-proliferative and senescent primary cells from 21M, a subpopulation of cells growing in colony-like structures was detected (Figure 1D). These colonies consisted of regularly shaped hexagonal cells that did not exhibit fibroblast-like morphology and were significantly smaller than the rest of 21M primary cells. Selective isolation of morphologically distinct colonies was performed, and the cells were continuously passaged, avoiding contamination with senescent cells by monitoring cellular morphology. These cells were designated HCEnC-21. In addition, identification, isolation, and continued passaging of a phenotypically distinct population of cells with highly uniform polygonal morphology were performed in primary cultures from 56- and 70-year-old donor corneas (Figure S1).

### Telomerase Increases Corneal Endothelial Proliferative Capacity without Loss of P53 Function

**Figure 2:**
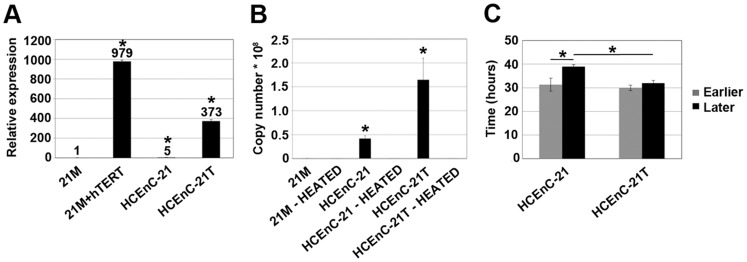
Telomerase expression, telomerase activity and cell doubling time. (A) Telomerase mRNA levels relative to 21M primary cells were detected by real-time PCR. Telomerase-transduced primary (21M+hTERT) and HCEnC-21 cells (HCEnC-21T) showed strongly elevated telomerase mRNA levels. Importantly, non-transduced HCEnC-21 cells also expressed significantly higher levels of telomerase mRNA than 21M primary cells. (B) Telomerase activity indicated as template copy number was measured in cell extracts using the telomeric repeat amplification protocol (TRAPeze RT). Heat-inactivated extracts served as negative controls. No specific telomerase activity was detected in 21M primary cells, while HCEnC-21T cell lysates contained strongly elevated telomerase activity. Note that HCEnC-21 intrinsically upregulated endogenous telomerase activity. (C) Cell doubling time of earlier (19–24) and later (32–41) passages of HCEnC-21 and HCEnC-21T. Cells were plated in 12-well plates at a density of 50,000 cells/well and counted after 2, 3, and 4 days using a hemocytometer. HCEnC-21T showed a higher proliferation rate compared to HCEnC-21 in long-term cultures. Error bars indicate mean ± SEM. *, P<0.05.

To investigate the role of hTERT overexpression on corneal endothelial cell proliferation, 21M primary and HCEnC-21 cells were transduced with hTERT mRNA (21M+hTERT and HCEnC-21T, respectively). Successful transduction was indicated by significantly increased hTERT mRNA levels in 21M+hTERT (979-fold, P = 0.00019) and HCEnC-21T (373-fold, P = 0.000017) cells (Figure 2A). Notably, HCEnC-21 cells expressed 5-fold (P = 0.00011) more hTERT mRNA than non-transduced 21M primary cells. However, despite high hTERT expression, 21M+hTERT cells were slow growing and developed typical signs of cellular senescence at the same passages as non-transduced cells (Figure 1I). Similarly, hTERT transduction of primary cells from the 56- and 70-year-old donors did not result in an extended life span. In contrast, both HCEnC-21 and HCEnC-21T cells have been proliferating for more than 70 passages (in 15 mo) without signs of senescence. Importantly, phase-contrast microscopy revealed that HCEnC-21 (Figures 1E–F and S2) and HCEnC-21T (Figures 1G–H and S2) continuously grew in monolayers in a contact-inhibited fashion and maintained their endothelial hexagonal morphology at all passages.

To confirm the functionality of hTERT detected in HCEnC-21 and HCEnC-21T, telomerase enzyme activity in cell lysates was assayed using the telomeric repeat amplification protocol (TRAP) (Figure 2B). Heat-inactivated cell extracts served as an internal negative control, since hTERT is a temperature-sensitive enzyme. While the telomerase activity of 21M primary cells was not significantly different from the respective heat-inactivated control, indicating the absence of telomerase activity in 21M, HCEnC-21T cells displayed a significant increase in telomerase activity of about 505-fold (P = 0.021) relative to 21M as deduced from the template copy numbers. Importantly, HCEnC-21 cell extracts exhibited a 110-fold (P = 0.00062) increase in telomerase activity compared to 21M, confirming the presence of endogenous telomerase activity in HCEnC-21.

To explore differences in the proliferative capacity, the cell doubling times (CDTs) of HCEnC-21 and HCEnC-21T cells were determined for earlier (19–24) and later (32–41) passages (Figure 2C). HCEnC-21 and HCEnC-21T cells had similar CDTs of around 30 hr at earlier passages. At later passages, HCEnC-21T cells maintained a high proliferation rate showing no significant difference in CDT, whereas HCEnC-21 cells exhibited a significantly increased CDT (39 hr, P = 0.04), indicating slower cell proliferation. This suggests that higher hTERT activity leads to a higher proliferation rate of HCEnC-21T cells compared to HCEnC-21 in long-term cell cultures.

**Figure 3:**
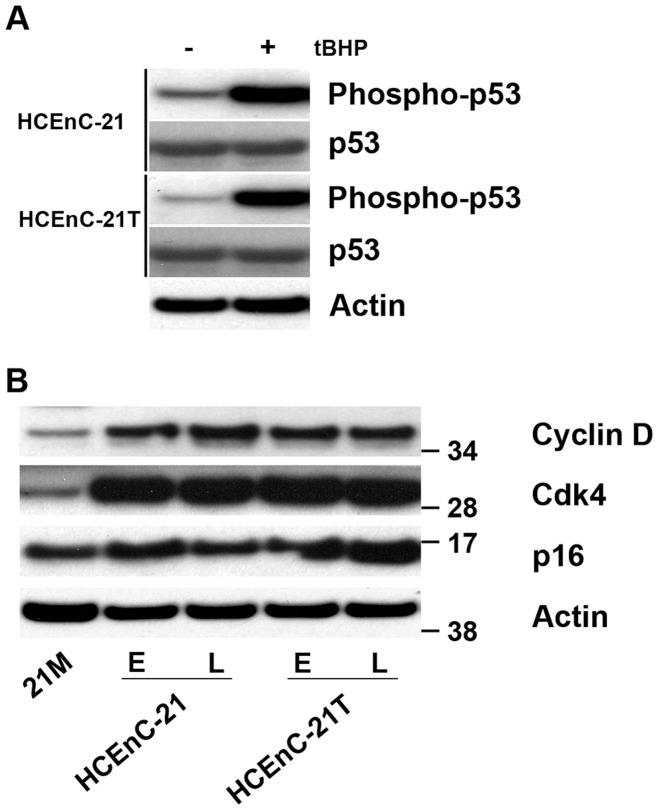
Synthesis of cyclin D, CDK4, p16^INK4^ and p53 in HCEnC-21 and HCEnC-21T. (A) Analysis of p53 functionality. Oxidative stress was induced in confluent monolayers of HCEnC-21 and HCEnC-21T cells using 50 µM *tert*-Butyl-hydroperoxide for 1 hr. Cell extracts were then separated by SDS-PAGE and immunoblotted with antibodies against p53, phospho-p53 (serine-15), and β-actin. P53 was detected in extracts of both HCEnC-21 and HCEnC-21T and levels of phospho-p53 (activated p53) were increased upon induction of oxidative stress. E: earlier passages <25. L: later passages >45. (B) Synthesis of G1 phase regulatory proteins. Cell extracts were run on SDS-polyacrylamide gels and immunoblotted with antibodies against cyclin D, CDK4, p16^INK4^ and β-actin. No changes in p16^INK4^ protein levels were detected. Cyclin D and CDK4 levels were increased in both, HCEnC-21 and HCEnC-21T compared to 21M.

Overexpression of oncogenes has so far been the only modality to enhance cell proliferation and prevent early senescence in corneal endothelial primary cells. Central to this approach is the inactivation of the p53 checkpoint, which, in turn, enhances the likelihood of oncogenic transformation. To evaluate whether such an oncogenic change is contributing to the proliferative capacity of HCEnC-21 and HCEnC-21T, synthesis of total p53 and activated p53, which is phosphorylated at serine-15 [20], [21], were investigated. HCEnC-21 and HCEnC-21T cells were grown to confluence and then treated with 50 µM *tert*-Butyl-hydroperoxide to induce oxidative stress. Figure 3A demonstrates that HCEnC-21 and HCEnC-21T cells synthesized p53 at baseline and that the levels of phosphorylated p53 (phospho-p53) became elevated after induction of oxidative stress. This suggests that the p53 pathway is functional and capable of sensing oxidative stress in HCEnC-21 and HCEnC-21T cells.

### CDK4 and Cyclin D Contribute to Increased HCEnC-21 and HCEnC-21T Proliferative Potential

HCEnCs are arrested in the G1 phase of the cell cycle in vivo and strongly tend to enter a senescence-related G1 arrest in vitro [22]. To elucidate the mechanism that underlies the increased growth potential of HCEnC-21 and HCEnC-21T cells compared to 21M primary cells, the steady-state protein levels of the key regulators that orchestrate the G1 phase and G1/S transition of the cell cycle were investigated. Figure 3B demonstrates that p16^INK4^ protein levels were similar in HCEnC-21 and HCEnC-21T as compared to 21M primary cells. However, CDK4 and cyclin D protein levels were elevated, and this increase was similar in earlier (<25) as well as later (>45) passages of HCEnC-21 and HCEnC-21T cells. These data suggest that upregulation of the cyclin D/CDK4 kinase, along with telomerase, plays a role in enhancing the proliferative capacity of this corneal endothelial cell subpopulation.

### HCEnC-21 and HCEnC-21T Synthesize Characteristic Corneal Endothelial Cell Markers

**Figure 4:**
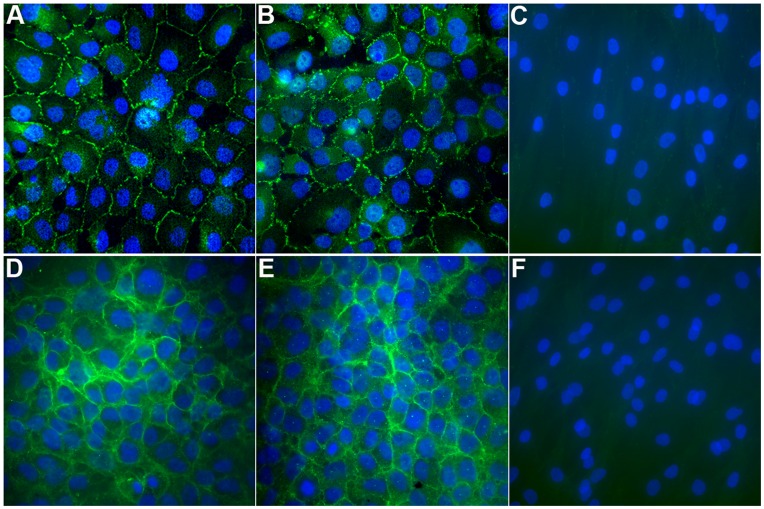
Immunofluorescence detection of ZO-1 and Na/K ATPase α1 in HCEnC-21 and HCEnC-21T. (A–C) Confluent monolayers of passage-13 HCEnC-21 (A) passage-14 HCEnC-21T (B) and passage-3 primary stromal fibroblasts (C) were fixed and labeled for ZO-1 (green) and nuclei (blue). (D–F) Confluent monolayers of HCEnC-21 (D), HCEnC-21T (E), and primary stromal fibroblasts (F) were fixed and labeled for Na/K ATPase α1 (green) and nuclei (blue). Note that ZO-1 and Na/K ATPase α1 were detected in HCEnC-21 and HCEnC-21T but not in stromal fibroblasts, and localized to the cell-cell contacts and plasma membrane, respectively. 400x.

**Figure 5:**
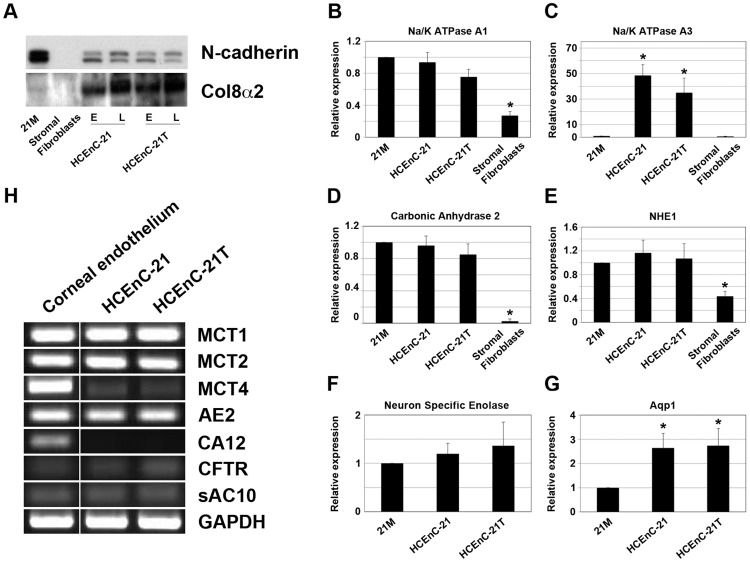
HCEnC-21 and HCEnC-21T cells express genes for ion pumping and typical corneal endothelial markers. (A) Cell extracts were separated by SDS-PAGE and immunoblotted for N-cadherin and collagen type 8 α2. 21M, HCEnC-21 and HCEnC-21T, but not stromal fibroblasts, synthesized N-cadherin and Col8α2. E: earlier passages <25. L: later passages >45. (B–G) Expression of (B) Na/K ATPase α1, (C) Na/K ATPase α3, (D) carbonic anhydrase 2 (CA2), (E) Na/H^+^ exchanger (NHE1), (F) neuron-specific enolase (NSE), and (G) aquaporin 1 (Aqp1) was detected by real-time PCR. Na/K ATPase α3 and Aqp1 showed increased expression; all other genes were expressed at similar levels relative to 21M primary cells. Stromal fibroblasts expressed significantly less Na/K ATPase α1 and α3, NHE1, and CA2 relative to HCEnC-21 and HCEnC-21T. (H) Monocarboxylate cotransporters (MCT1, -2, and -4), anion exchanger 2 (AE2), carbonic anhydrase 12 (CA12), cystic fibrosis transmembrane conductance regulator (CFTR), soluble adenylyl cyclase 10 (sAC10), and GAPDH were detected by RT PCR. All genes, except for MCT4 and CA12, showed similar expression in HCEnC-21 and HCEnC-21T compared to corneal endothelial tissue. Error bars indicate mean ± SEM. *, p<0.05.

Cell-cell contacts between corneal endothelial cells are the fundamental basis for the barrier function of HCEn and consist of tight junctions as well as cadherin-based adherens junctions [23]. ZO-1, an essential component of tight junctions and one of the key markers for HCEn, was detected by immunofluorescence staining of HCEnC-21 and HCEnC-21T monolayers. Primary stromal fibroblasts were used as a biological negative control. Immunostaining with ZO-1 was detected in both HCEnC-21 (passage 13, Figure 4A) and HCEnC-21T (passage 14, Figure 4B) cells and localized to the cell-cell junctions, whereas no staining of stromal fibroblasts was detected (Figure 4C). Similar staining with ZO-1 was observed in later passages of HCEnC-21 (passage 32) and HCEnC-21T (passage 46) cells (Figure S3). The ZO-1 immunostaining demonstrates the typical polygonal morphology of HCEnC-21 and HCEnC-21T cells and suggests the formation of tight junctions between neighboring cells. Additionally, N-cadherin, a key component of adherens junctions, was detected in cell extracts from earlier (<25) and later (>45) passages of HCEnC-21 and HCEnC-21T cells by Western blotting (Figure 5A). No N-cadherin was present in lysates from stromal fibroblasts.

Collagen type 8 is synthesized by HCEnCs and is the major component of Descemets membrane, the corneal endothelial basement membrane [24]. Figure 5A shows that the collagen type 8 α2 chain is detected in corneal endothelial primary cells as well as in HCEnC-21 and HCEnC-21T cells, but not in stromal fibroblasts, demonstrating the presence of another major marker for HCEn in HCEnC-21 and HCEnC-21T cells.

### HCEnC-21 and HCEnC-21T Express Typical Corneal Endothelial Ion Transporters

To further study the corneal endothelial-specific phenotype of HCEnC-21 and HCEnC-21T cells, expression of 14 different ion transporter genes was investigated (Figure 5B–H). Na/K ATPase is one of the most important ion transporters responsible for creating an ionic gradient across the corneal endothelial basolateral membrane and is composed of α and β subunits [25], [26]. Both α and β subunits exist in different isoforms that are synthesized in a tissue-specific manner [27]. HCEn is known to distinctly express the α1 and α3 isoforms, which are responsible for hydrolyzing ATP and ion transportation [28]. Real-time PCR showed similar expression of the Na/K ATPase α1 subunit (Figure 5B) and upregulation of the Na/K ATPase α3 subunit (Figure 5C) in HCEnC-21 and HCEnC-21T cells compared to 21M primary cells. Immunofluorescence staining confirmed that the Na/K ATPase α1 subunit was highly abundant in HCEnC-21 (Figure 4D) and HCEnC-21T (Figure 4E) cells, and mainly localized to the basolateral membrane. In contrast, Na/K ATPase α1 was not detected in primary stromal fibroblasts (Figure 4F), pointing out the specificity of the HCEnC-21 and HCEnC-21T cell phenotype.

Monocarboxylate cotransporters (MCTs) 1, 2, and 4 facilitate lactate transport across the HCEn by employing a lactate-H^+^ cotransport mechanism [29]. MCT lactate transport activity is increased by interaction with carbonic anhydrase 2 (CA2) [30], [31], [32] and is further augmented by the Na/H^+^ exchanger 1 (NHE1) [29]. Although MCT4 expression was decreased in HCEnC-21 and HCEnC-21T cells, MCT1 and MCT2 were expressed at levels similar to those in corneal endothelial tissue (Figure 5H). Figures 5D and E illustrate that CA2 and NHE1 mRNA levels, respectively, were similar in HCEnC-21 and HCEnC-21T as compared to levels in 21M primary cells. Additionally, neuron-specific enolase (NSE), anion exchanger 2 (AE2), cystic fibrosis transmembrane conductance regulator (CFTR), soluble adenylyl cyclase 10 (sAC10), and aquaporin 1 (Aqp1) were expressed at similar or slightly increased levels compared to either 21M primary cells or corneal endothelial tissue (Figure 5F–H). These results indicate that the expression of ion transporters essential for corneal endothelial pump activity is retained at normal levels in HCEnC-21 and HCEnC-21T cells. Comparison with stromal fibroblasts supports the distinct corneal endothelial expression profile of HCEnC-21 and HCEnC-21T cells.

### Functional Characterization of Barrier Integrity and Ion Pump Function

**Figure 6:**
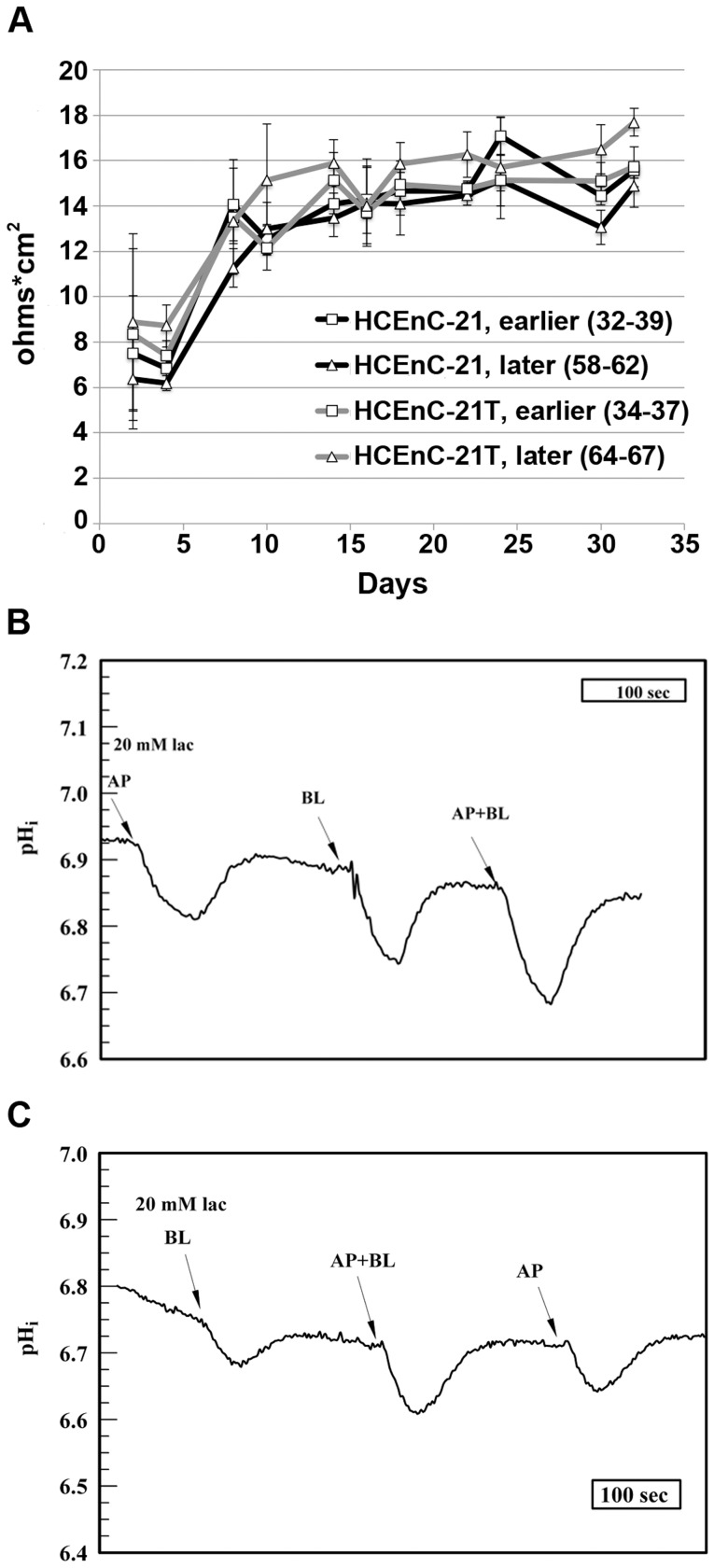
HCEnC-21 and HCEnC-21T retain typical corneal endothelial barrier integrity and pump function. (A) Cells were plated in 12-well transwell inserts (0.4 µm) at a density of 100,000 cells per transwell and transendothelial resistance (TER) was measured every 2 or 4 days over the course of 4.5 wk. Note that earlier (32–39) and later (58–67) passages of both HCEnC-21 and HCEnC-21T established a typical corneal endothelial barrier of 15 Ω*cm^2^ after about 2 wk and maintained this barrier for 2.5 more wk. (B,C) HCEnC-21 (B) and HCEnC-21T (C) were grown to confluence (3,000 cells/cm^2^) in perfusion chambers and 20 mM lactate was applied to their apical, basolateral or both cell membranes. Facilitated lactate uptake is H^+^ coupled and was measured indirectly by detecting intracellular pH changes using the BCECF-AM fluorescent dye. Temporal acidification of both HCEnC-21 and HCEnC-21T was observed after lactate pulses to the apical and basolateral membrane. Error bars indicate mean ± SEM. *, P<0.05.

The corneal endothelial cell-cell junctions are known to form a “leaky” barrier, which allows paracellular nutrient diffusion into the cornea. In order to measure the barrier integrity of HCEnC-21 and HCEnC-21T cells, transendothelial resistance (TER) was determined. HCEn has been shown to establish a TER of 15–25 Ω*cm^2^ in vitro [33]. Figure 6A depicts the TER measured in HCEnC-21 and HCEnC-21T cells over the course of 4.5 wk. After a steep initial increase during the first 10 days, TER gradually increased for the next 3 wk. After 3–4 wk, peak TER values measured between 15–18 Ω*cm^2^. No significant differences were detected between HCEnC-21 and HCEnC-21T, as well as between earlier (32–39) and later (49–58) passages. These results indicate that HCEnC-21 and HCEnC-21T establish and maintain a typical TER resembling the distinct corneal endothelial barrier integrity.

Lactate is a major waste product that is continuously eliminated from the corneal stroma across the corneal endothelium. Failure to remove lactate results in corneal edema and loss of visual acuity [34]. Cellular lactate uptake can be indirectly measured by detecting changes in the intracellular pH, using a pH-sensitive fluorescent dye [29]. As shown in Figure 6B–C, the intracellular pH of HCEnC-21 and HCEnC-21T cells before addition of lactate was between 6.8 and 7.0. Both HCEnC-21 and HCEnC-21T cells were instantly acidified upon addition of lactate to the apical, basolateral, or both cell membranes by about 0.1 on the pH scale. After 1 min, the excess H^+^ was cleared and the pH recovered to normal levels. This data suggest that HCEnC-21 and HCEnC-21T retain their ability to actively take up lactate in an H^+^-coupled manner, indicating typical corneal endothelial ion transport characteristics.

## Discussion

Primary HCEnCs have very limited proliferative potential and life span, rendering them of limited use in the study of endothelial cell biology and development of cell-based therapies for corneal conditions. This study provides evidence that hTERT overexpression generates a mitotically stable, homogenous population of human corneal endothelial cells that retain functional competence. We have detected that there is phenotypic heterogeneity of HCEn in vitro and that increased self-renewal competence of a subpopulation of endothelial cells could be related to their intrinsic upregulation of telomerase activity along with increased synthesis of cyclin D and CDK4. Additional overexpression of hTERT generates a stable homogeneous population of cells that have increased mitotic capacity over non-transduced counterparts and exhibit a functional and morphologic phenotype characteristic of HCEn in vivo.

The corneal endothelial cell cycle is tightly regulated by CDKs and their binding partners cyclins, as well as cyclin-dependent kinase inhibitors, such as p16^INK4^ and p21^CIP1^. The progression through G1 phase is driven by increased levels of cyclin D bound to CDK4 that together activate genes involved in G1/S transition and inactivate cell cycle inhibitors via posttranslational modification. Primary corneal endothelial cells readily enter cellular (replicative) senescence, a process that limits cell division [35], [36], and has been attributed to the activation of p53-target genes and upregulation of p21^CIP1^ and p16^INK4^ [37]. Replicative senescence of HCEn in vitro is highly dependent on donor age, with cells from older donors undergoing cell cycle arrest at earlier passages and expressing higher levels of p16^INK4^ and p21^CIP1^ [38]. In our study, HCEnC-21 and HCEnC-21T exhibited p16^INK4^ levels similar to those of primary cells, while cyclin D and CDK4 synthesis was upregulated, indicating that these factors are crucial in bypassing control mechanisms of cellular senescence, while maintaining an endothelial phenotype. Since the life span of HCEnC-21 and HCEnC-21T cells was extended without overexpression of oncogenes (such as HPV E6/E7 or SV40 large T antigen), the cells maintained baseline synthesis of p53 and readily upregulated phospho-p53 during oxidative stress, indicating the presence of pathways that control normal cellular stress response. This is advantageous for the study of molecular mechanisms of endothelial diseases such as Fuchs endothelial corneal dystrophy, which is one of the major causes for corneal transplantation in the elderly population and is caused by p53-dependent apoptosis of HCEn [11]. The disadvantage of our approach is that reliance on hTERT immortalization of a specific subpopulation of corneal endothelial cells might, in turn, diminish the likelihood of successful immortalization of endothelial cells that otherwise could have been achieved more efficiently by oncogenic transformation.

The majority of primary cells from the 21M donor senesced at passage 9 and was not responsive to hTERT immortalization (Figure 1I), consistent with a recent report indicating that hTERT expression alone does not overcome senescence-related growth arrest in primary HCEnCs [39]. However, the subpopulation of primary cells designated HCEnC-21 did not show any signs of cellular senescence for more than 70 passages and was conducive to retrovirally mediated hTERT immortalization. This phenotypic diversity of cell culture was also observed in 56-year-old and 70-year-old donors (Figure S1), indicating the novel finding that HCEn consists of a heterogeneous population of cells that display various degrees of replicative competence. Therefore, we found a repeated presence of a subpopulation of cells growing in colony-like structures in several different primary cultures, indicating that this phenomenon is reproducible in cultures taken from different donors. In support of our findings, a previous study on bovine corneal endothelium detected a subset of cells with robust proliferative potential that reside interspersed among cells of limited proliferative potential, providing the basis for the possible existence of corneal endothelial progenitor cells [40]. Here we provide the first report of a similar phenomenon in HCEn, although more studies are needed to determine whether these highly replicative cells represent human endothelial progenitor cells.

Human TERT is the enzyme responsible for synthesizing and maintaining telomeres; however, the hTERT gene is turned off after birth in the majority of cell types, except for some progenitor and stem cells [41]. Absence of active telomerase leads to telomere shortening and erosion, which, in turn, determines the finite life span of proliferating cells [42]. Similarly, HCEnCs do not possess any telomerase activity either in vivo or in vitro [43], purportedly leading to cellular senescence seen in primary cultures. Interestingly, in the current study, HCEnC-21 exhibited higher levels of telomerase mRNA and activity than primary cells and, like HCEnC-21T, did not senesce. Notably, the CDTs of early-passage HCEnC-21 and HCEnC-21T (passages 19–24), which were approximately 30 hr, were relatively short compared to the CDT of primary HCEnCs, which doubled once in approximately 45 hr [44]. These data support the increased proliferative potential of HCEnC-21 and HCEnC-21T. At later passages (32–41), HCEnC-21T cells were able to preserve their high proliferative rate, while HCEnC-21 displayed slower proliferation, indicating that the significantly enhanced hTERT activity accounted for the superior proliferative rate of HCEnC-21T.

The major functions of HCEn are to provide a leaky barrier between the aqueous humor and stroma and to counteract the stromal (corneal) swelling pressure by active ion transport. The barrier function of HCEnCs is dependent on their ability to form tight junctions [33], [45], [46]. Our data show that ZO-1, a key component of tight junctions, is synthesized in HCEnC-21 and HCEnC-21T cells and localizes to the cell-cell boundaries. This staining pattern is expected for functional tight junctions and resembles the staining patterns seen in vivo [45], [46]. In addition, N-cadherin, which is part of the tight junction-associated adherens junctions, was produced in HCEnC-21 and HCEnC-21T cells as seen in corneal endothelial cells in vivo [47]. The functionality of the junctional complexes was confirmed by measurement of the TER. The TER of HCEnC-21 and HCEnC-21T cells reached 15–18 Ω*cm^2,^ which is typical for the leaky barrier of HCEnCs [33]. Regarding the corneal endothelial pump function, we demonstrated that HCEnC-21 and HCEnC-21T cells accumulate Na/K ATPase α1 in their plasma membranes and that they express a variety of ion transporters normally found in HCEn [48]. It was recently established that coupled lactate and proton pumping is an essential component of the corneal endothelial pump [29], and the functional analysis of lactate transport revealed that HCEnC-21 and HCEnC-21T cells actively pump lactate across their cell membranes as evidenced by corresponding pH changes.

This study is significant because it demonstrates that propagation of HCEn in vitro can be achieved through telomerase overexpression, while desired hexagonal morphology, marker gene expression, and corneal endothelial functionality are retained. Since the inability to regenerate endothelium remains a major challenge in ophthalmology, the possibility of identifying a population of HCEnCs with self-renewal competence and stimulating its growth potential in vivo could generate novel regenerative therapies, hopefully reducing the need for corneal transplantation.

## Materials and Methods

### Ethics Statement

This study was approved by the institutional review board of Schepens Eye Research Institute. Donor corneas were obtained from the eye bank National Disease Research Interchange (NDRI; Philadelphia, PA).

### Cell Culture

Donor corneas were obtained according to the exclusion criteria reported previously [44] and were maintained in corneal storage medium (Optisol™; Chiron Ophthalmics, Inc.; Irvine, CA) at 4°C until immediately before isolation of corneal endothelial cells. Primary cells were cultured according to previously published methods [49] with minor modifications. Briefly, after dissection of Descemets membrane with intact endothelium and overnight stabilization in complete medium (OptiMEM-I®; Invitrogen; Carlsbad, CA), 8% FBS (Hyclone Laboratories, Inc.; Logan UT), EGF 5 ng/mL (Millipore; Billerica, MA), pituitary extract 100 µg/mL (Hyclone Laboratories), calcium chloride 200 mg/L, 0.08% chondroitin sulfate (Sigma-Aldrich; St. Louis, MA), gentamicin 50 µg/mL, and antibiotic/antimycotic solution diluted 1∶100 (Invitrogen), the strips were incubated in 0.02% EDTA solution (Sigma-Aldrich) at 37°C for 1 hr and mechanically disrupted by trituration. Cell suspensions were plated in 12-well tissue culture plates precoated with undiluted FNC Coating Mix® (AthenaES; Baltimore MD). Subculturing of corneal endothelial cell cultures was done using 0.05% trypsin (Invitrogen) for 5 min at 37°C. Phase-contrast microscopy was employed to detect cell morphologic changes over time using a Nikon Eclipse TS100 microscope with a Diagnostic Instruments 11.2 Color Magic digital camera (Nikon; Tokyo, Japan).

### Retroviral Transduction of HCEnCs

293GPG cells [50] were grown on 15-cm culture dishes in DMEM growth medium (Invitrogen) (10% heat-inactivated FBS (Hyclone Laboratories), 50 U/mL penicillin-streptomycin (Invitrogen), 1 µg/mL tetracycline, 2 µg/mL puromycin (Sigma-Aldrich), and 0.3 mg/mL Geneticin G418® (Sigma-Aldrich) and transfected with pBABE-puro-hTERT (plasmid 1771, Addgene; http://www.addgene.org/) using Lipofectamine® 2000 (Invitrogen) at 80% confluence. Reduced growth medium without tetracycline, puromycin, and Geneticin was added after 18 hr, and virus-containing supernatant was collected from days 2 to 6. Concentrated virus particles were stored as single-use aliquots at -80°C in sterile TNE buffer (50 mM Tris (pH 7.8), 130 mM NaCl, and 1 mM EDTA (Sigma-Aldrich)).

Primary cells were plated in 6-well plates or T75 culture flasks and grown to 60% confluence. Fresh medium containing 8 µg/mL polybrene (Millipore), as well as either 50 µl (6-well) or 150 µl (T75) concentrated virus suspension, was added to the cells every 24 hr for 5 consecutive days. Cells were then selected against 1 µg/mL puromycin (Sigma-Aldrich) for 7 days, and resistant cells were expanded and subcultured in normal growth medium.

### Cell Doubling Time (CDT)

HCEnC-21 and HCEnC-21T cells were plated in 12-well plates at a density of 50,000 cells per well. Medium was changed at day 2. Cells were trypsinized and counted using a Hemocytometer (Hausser Scientific; Horsham, PA), after 2, 3, and 4 days. Cell numbers were determined for duplicate wells per time point. Two to four different passages were independently analyzed per group, and cell type and results were averaged for data analysis.

### Real-time and Reverse Transcription (RT) PCR

Total RNA from cell cultures was isolated using the RNeasy kits (Qiagen; Valencia, CA) or TRIzol® (Invitrogen). Reverse transcription was carried out using a commercially available kit according to the manufacturer’s protocol. TaqMan® primers for β-2 microglobulin (B2M), hTERT, Na/K ATPase α1 and α3, NHE1, CA2, Aqp1, and NSE were obtained from Applied Biosystems (Foster City, PA). Real-time PCR reactions were set up in triplicate (Probe Fast master mix; Kapa Biosystems; Woburn, MA), and every gene was detected in at least 3 different passages of HCEnC-21 and HCEnC-21T cells, as well as in 2 passages (<6) of 21M primary cells and stromal fibroblasts. PCR was performed in a Mastercycler Realplex2 (Eppendorf; Hamburg, Germany). For data analysis, results were averaged, SEM was calculated, and the comparative Ct method was performed using B2M as the calibrator. Conventional PCR was performed in a MyCycler Thermal cycler (Bio-Rad; Hercules, CA) following the AmpliTaq® 360 DNA Polymerase protocol (Applied Biosystems) using primers specific to MCT1, −2, and −4, AE2, CA12, CFTR, sAC10, and glyceraldehyde-3-phosphate dehydrogenase (GAPDH). The nucleotide sequences of these primers are listed in Table 1. cDNA (1 µl) was added into a 25 µl reaction that underwent 30 cycles of amplification. PCR products (10 µl) were examined on 1.2% agarose gels stained with ethidium bromide. No-template-controls were performed at the reverse transcription and PCR steps and served as negative controls.

**Table 1:** Primer Sequences for RT-PCR.

Gene ID	Primer Name	Sequence (5′–3′)	Position	cDNA (bp)
hMCT1 NM_003051.3	hMCT1-F	AGCGAAGTGTCATGGATATCCTCC	529–934	406
	hMCT1-R	CAACACAGCAGTTTAGTAGCAAGC		
hMCT2 NM_004731.3	hMCT2-F	CCCACATGTACACAGAGTATCTGG	2075–2419	345
hMCT2 NM_004731.3	hMCT2-R	AGGGTTCTATTCTCTAGCACCAGG	2075–2419	345
hMCT4 NM_001042423.1	hMCT4-F	GTCAGTGTCTTCTTCAAGGAGCTC	242–540	299
hMCT4 NM_001042423.1	hMCT4-R	AAGTAGCGGTTCAGCATGATGAGC	242–540	299
hAE2 U62531.1	hAE2-F	CAGTTCTTTCTCCGAGAGGATGAC	631–1001	371
hAE2 U62531.1	hAE2-R	TGACTCTTCATGAGGTCTAGGTCG	631–1001	371
hCA12 BC023981.1	hCA12-F	TTGGCATCTGTATTGTGGTGGTGG	1071–1431	361
hCA12 BC023981.1	hCA12-R	CAGCTTTGAATTCCTGCTGCTTGG	1071–1431	361
hCFTR NM_000492.3	hCFTR-F	TGGTGATGACAGCCTCTTCTTCAG	1401–1737	337
hCFTR NM_000492.3	hCFTR-R	CTCTGCAAACTTGGAGATGTCCTC	1401–1737	337
hsAC10 NM_018417.4	hsAC10-F	TGTCTTGACCTCAATGTGAGCTGC	2426–2772	347
hsAC10 NM_018417.4	hsAC10-R	GAAAGTCTCATGCTATCCAGCTGG	2426–2772	347
hGAPDH NM_002046.3	hGAPDH-F	TTCCACCCATGGCAAATTCCATGG	252–554	303
hGAPDH NM_002046.3	hGAPDH-R	GAGGCATTGCTGATGATCTTGAGG	252–554	303

### Telomeric Repeat Amplification Protocol (TRAP)

TRAP assays were performed using the TRAPeze RT kit (Millipore) according to the manufacture’s protocol. Briefly, cells were harvested, washed with PBS, and stored at −80°C until use. After thawing, cells were immediately extracted with CHAPS lysis buffer (Millipore) and total protein concentration was determined using the BCA™ Reducing Agent Compatible Assay (Pierce; Rockfort, IL) TRAP reactions were performed with 1 µg protein (samples and positive control), and results were transformed into template copy numbers based on TSR8 standard curves. Mean values were calculated from a minimum of 3 different passages per cell type run in 2 independent experiments.

### Gel Electrophoresis and Western Blotting

Between 0.5–2*10^6^ cells were lysed in 200 µl RIPA buffer containing HALT protease and phosphatase inhibitors (both Thermo Scientific®; Rockfort, IL) for 30 min on ice. Cell lysates were passed 6 times through a 26-gauge needle and centrifuged at 15,000×g and 4°C for 15 min. The supernatant (180 µl) was transferred into a new tube and the BCA assay (Pierce) was used to determine total protein concentration.

Equal amounts of protein were loaded on 10% Bis-Tris gels for SDS-PAGE. Proteins were then electrophoretically transferred to a polyvinylidene difluoride membrane (Millipore), and nonspecific binding was blocked by incubation in 5% nonfat milk diluted in tris-buffered saline containing 0.1% Tween-20 (TBST) for 1 hr at room temperature. Membranes were incubated overnight on a horizontal shaker at 4°C, with primary antibodies diluted in blocking buffer. Primary antibodies used were collagen type 8 α2 (1∶100), N-cadherin (1∶200), p53 (1∶200), and p16^INK4^ (1∶100) (all Santa Cruz Biotechnology; Santa Cruz, CA), cyclin D (1∶500; Millipore), CDK4 (1∶200; Thermo Scientific®), Phospho-p53 (1∶5000; Cell Signaling Technology; Danvers, MA) and mouse monoclonal β-actin (1∶10000, Sigma Aldrich). Blots were rinsed with TBST 3 times for 10 min each and exposed to HRPconjugated donkey anti-mouse or -rabbit IgG for 1 hr at room temperature. All secondary antibodies were obtained from Jackson ImmunoResearch Laboratories, Inc. (West Grove, PA) and diluted 1∶2000 in blocking buffer. After washing 3 times in TBST, proteins were detected with SuperSignal® Pico or Femto chemiluminescent substrate (Thermo Scientific). A minimum of 2 different passages were analyzed per group and cell type.

### Immunocytochemistry

Cells were plated in FNC-coated (AthenaES) 2-well chamber slides and grown to confluence. Cells were carefully washed with PBS and fixed in 4% PFA, 100% −20°C-cold methanol, or 100% −20°C-cold acetone, depending on the primary antibody used. After washing twice with PBS, PFA-fixed cells were permeabilized for 5 min in 0.2% Triton™ X-100 (Sigma-Aldrich) in PBS. Fixed cells were blocked in 1% BSA in PBS for 1 hr at room temperature, before primary antibody was added at 4°C over night. Primary antibodies used were mouse and rabbit anti-ZO-1 (both 1∶100; Invitrogen) and mouse anti-Na ATPase α1 (1∶100; Abcam, La Jolla, CA). Cells were washed twice with PBS and were incubated with FITC-labeled secondary antibodies (1∶100; Jackson ImmunoResearch Laboratories) in blocking buffer for 1 hr at room temperature. After washing 3 times in PBS, slides were mounted in medium containing DAPI (Vectashield®; Vector Laboratories; Burlingame, CA). Fluorescence was visualized on a Nikon Eclipse E-800 fluorescence microscope equipped with a spot digital camera.

### Transendothelial Resistance (TER)

HCEnC-21 and HCEnC-21T cells were plated in FNC-coated, 12-well transwell inserts (1.12 cm^2^ growth area, 0.4 µm pore size) at a density of 100,000 cells per transwell. Medium was changed every other day. TER was measured every 2–4 days using the EVOM2 Epithelial VoltOhmMeter (WPI; Sarasota, FL) over the course of 4.5 wk. Immortalized human corneal epithelial (HCLE) cells were used as a positive control, whereas an empty FNC-coated (AthenaES) transwell served as a background control. Two to three different passages per group per cell type were analyzed. For data analysis, TER values for every time point were averaged and the SEM was calculated.

### Measurement of Proton-coupled Lactate Uptake

Lactate-dependent pH-tracing measurements were conducted as previously published [29], with minor modifications. In brief, HCEnC-21 and HCEnC-21T cells were cultured on a 0.2 µm pore size, rigid Anodisc filter (Whatman; Kent, UK) and incubated in bicarbonate-free Ringer solution containing 10 µM of the pH-sensitive fluorescent dye 2′7′-bis(2-carboxyethel)-5(6)-carboxyfluorescein acetoxymethyl ester (BCECF-AM) at room temperature for 30 min. Anodisc filters were placed in a double-sided perfusion chamber [51] and lactate (20 mM) was applied for 20–25 sec to the apical and basolateral compartments by gravity flow (approximately 0.5 ml per min). The temperature was constant at 37°C throughout the measurement. Fluorescence was excited at both 495±10 and 440±10 nm, and emission was detected at 520–550 nm. Emission ratios were calculated for excitation at 495 and 440 nm (495/440). Ratios obtained at 1 Hz were calibrated against pH by the high-K^+^-nigericin technique [51], [52].

### Statistics

Statistical analysis was performed using the Student’s unpaired t-test with the help of a free online calculator (StudentsTTest.com). P<0.05 was considered significant.

## Supporting Information

Figure S1 **Existence of distinct subpopulations of cells in primary cultures from older donors.** (A,B) Phase contrast micrographs of primary cells from 56-year-old donor at passage 3. (C,D) Phase contrast micrographs of primary cells from 70-year-old donor at passage 1. Note that uniform subpopulations with polygonal cells growing in monolayers (B,D) were detected among non-dividing cells displaying signs of senescence (A,C) in primary cultures of both older donors. 200x.
(TIF)

Figure S2 **Morphology of subconfluent HCEnC-21 and HCEnC-21T cells.** Phase-contrast micrographs of HCEnC-21 (A) and telomerase-transduced HCEnC-21 (HCEnC-21T; (B) cells in the subconfluent state.
(TIF)

Figure S3 **Immunofluorescence detection of ZO-1 in later passages of HCEnC-21 and HCEnC-21T.** (A) Confluent monolayers of passage-32 HCEnC-21 and (B) passage-46 HCEnC-21T cells were fixed and labeled for ZO-1 (green) and nuclei (blue). (C) Representative example of the secondary antibody only controls showing no antibody binding. Note that ZO-1 in later passage cells localized to the cell-cell contacts as seen in earlier passage cells. 400x.
(TIF)
