# Supplementary figures and images for: Telomerase Immortalization of Human Corneal Endothelial Cells Yields Functional Hexagonal Monolayers
Source: PLoS One. 2012 Dec 21;7(12):e51427. doi: 10.1371/journal.pone.0051427 (PMC3528758; doi:10.1371/journal.pone.0051427)

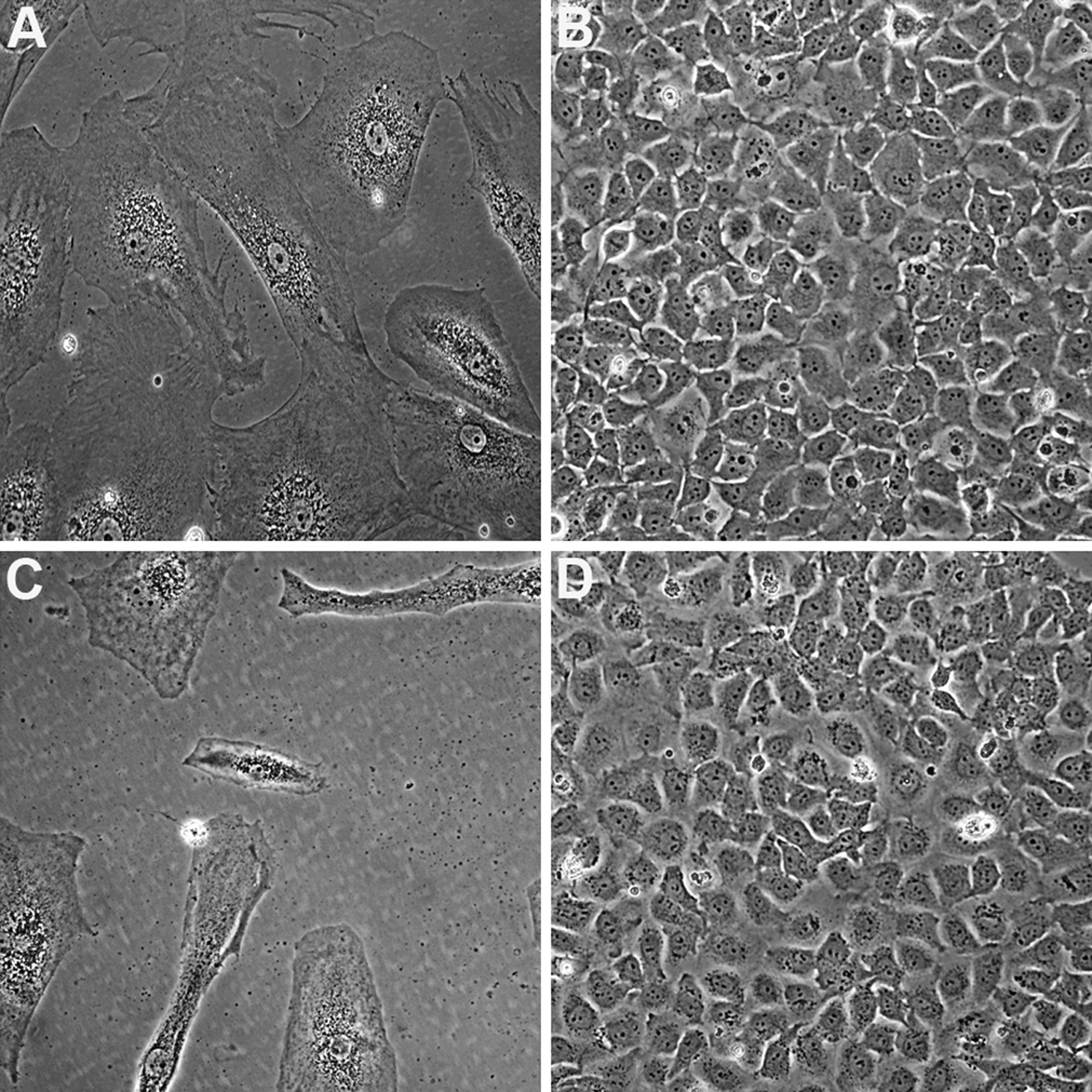

Supplement: Figure S1 — Existence of distinct subpopulations of cells in primary cultures from older donors. (A,B) Phase contrast micrographs of primary cells from 56-year-old donor at passage 3. (C,D) Phase contrast micrographs of primary cells from 70-year-old donor at passage 1. Note that uniform subpopulations with polygonal cells growing in monolayers (B,D) were detected among non-dividing cells displaying signs of senescence (A,C) in primary cultures of both older donors. 200x. (TIF) [file pone.0051427.s001.tif]

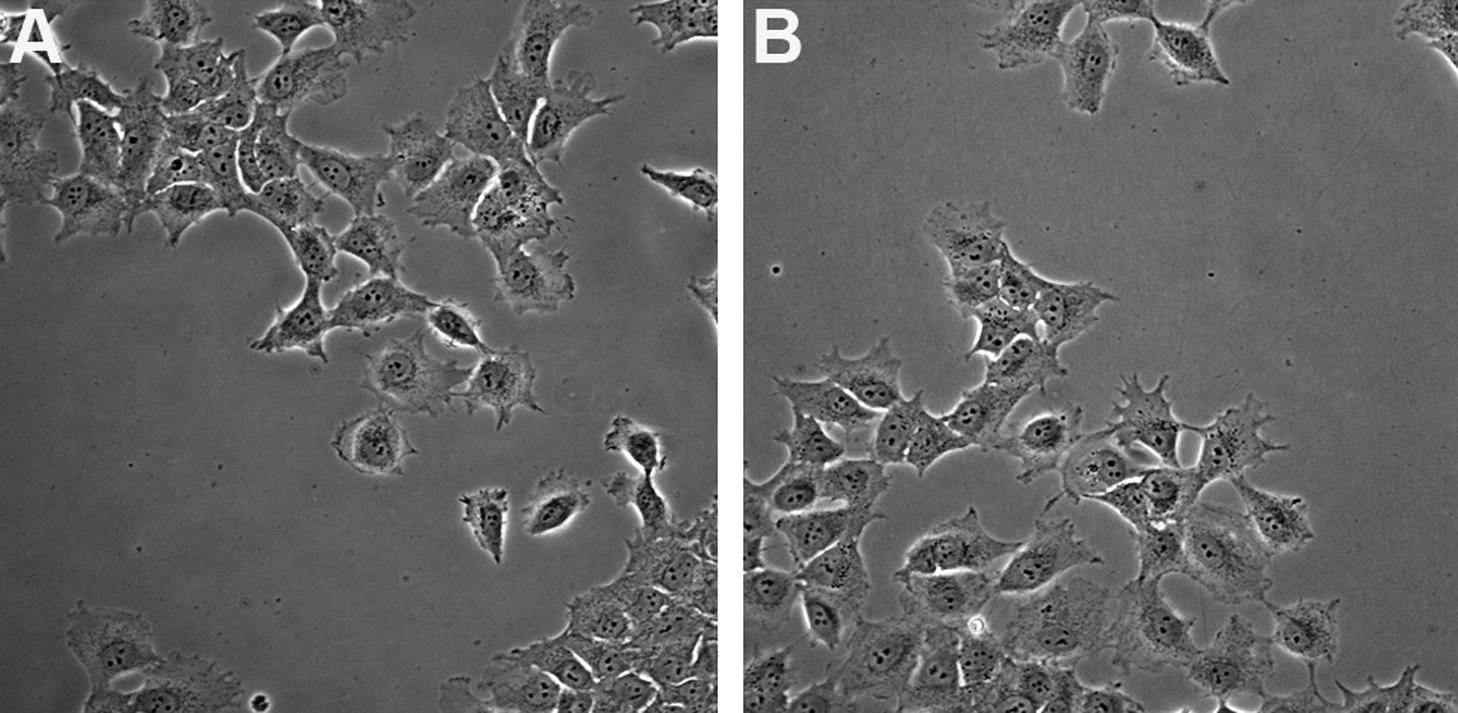

Supplement: Figure S2 — Morphology of subconfluent HCEnC-21 and HCEnC-21T cells. Phase-contrast micrographs of HCEnC-21 (A) and telomerase-transduced HCEnC-21 (HCEnC-21T; (B) cells in the subconfluent state. (TIF) [file pone.0051427.s002.tif]

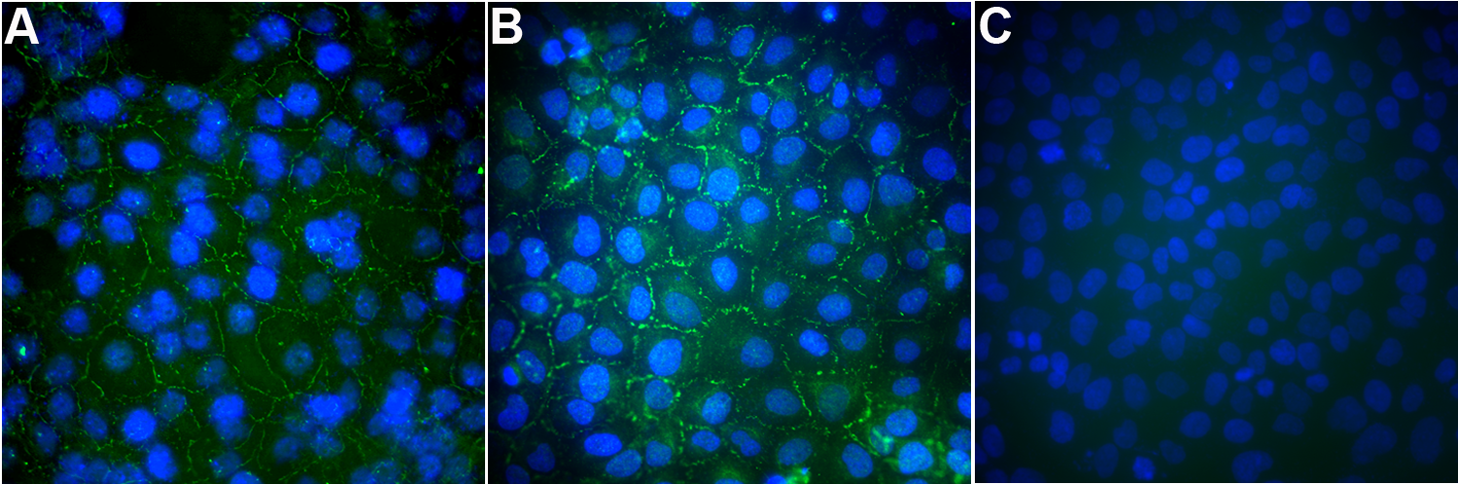

Supplement: Figure S3 — Immunofluorescence detection of ZO-1 in later passages of HCEnC-21 and HCEnC-21T. (A) Confluent monolayers of passage-32 HCEnC-21 and (B) passage-46 HCEnC-21T cells were fixed and labeled for ZO-1 (green) and nuclei (blue). (C) Representative example of the secondary antibody only controls showing no antibody binding. Note that ZO-1 in later passage cells localized to the cell-cell contacts as seen in earlier passage cells. 400x. (TIF) [file pone.0051427.s003.tif]
